# *MMP8* and *MMP9* gene polymorphisms were associated with breast cancer risk in a Chinese Han population

**DOI:** 10.1038/s41598-018-31664-3

**Published:** 2018-09-07

**Authors:** Kai Wang, Yi Zhou, Gang Li, Xinli Wen, Yuan Kou, Jiao Yu, Haifeng He, Qian Zhao, Feng Xue, Jin Wang, Xinhan Zhao

**Affiliations:** 1 Department of Internal Medicine Oncology, the First Affiliated Hospital of Xi’an Jiaotong University, Xi’an, Shaanxi 710061 China; 2 The Second Department of Spleen and Stomach, the Hospital of Traditional Chinese Medicine of Shaanxi province, Xi’an, Shaanxi 710063 China; 3 The Center for Medical Imaging, the Hospital of Traditional Chinese Medicine of Shaanxi province, Xi’an, Shaanxi 710063 China; 4 The Second Department of Thoracic Surgery, the First Affiliated Hospital of Xi’an Jiaotong University, Xi’an, Shaanxi 710061 China; 5 Department of encephalopathy, the Hospital of Traditional Chinese Medicine of Shaanxi province, Xi’an, Shaanxi 710063 China

## Abstract

Matrix metalloproteinases (MMPs) are a group of zinc-dependent endopeptidases that can breakdown almost all extracellular matrix components. *MMP8* and *MMP9* have been shown to be associated with breast cancer (BC) risk in European and American populations. However, few studies have focused on the polymorphisms of *MMP8* and *MMP9* in Chinese Han BC patients. We investigated nine single nucleotide polymorphisms (SNPs) in 571 BC cases and 578 controls to evaluating their association with risk of BC. The frequency of the “A” allele of rs3787268 was significantly lower in BC cases than in controls (*P* = 0.025). In the genetic model analysis, the minor allele “T” of rs11225394 in *MMP8* was associated with increased risk of BC under the recessive model (*P* = 0.019), and the minor allele “A” of rs3787268 was associated with decreased risk of BC under the dominant model (*P* = 0.014). Additionally, the haplotype “AGTCA” constructed by rs3740938, rs2012390, rs1940475, rs11225394, and rs11225395 and the haplotype “CCG” constructed by rs3918249, rs3918254 and rs3787268 were associated with increased risk of BC (*P* < 0.05). Our data showed that polymorphisms of *MMP8* and *MMP9* may be associated with BC risk in the Chinese Han population.

## Introduction

Breast cancer (BC) is the most frequently diagnosed cancer among females, and its incidence and mortality rate have continued to increase in recent years worldwide^1^. Identification of risk factors and early detection are important to clinical treatment for BC. BC is a multifactorial disease that is influenced by both genetic and environmental factors^2^. The environmental factors mainly include reproductive patterns, obesity, physical activity^3^ and some other lifestyle factors. However, hereditary factors account for a higher proportion of the BC burden. Genome-wide association studies (GWAS) have reported several susceptible single nucleotide polymorphisms (SNPs) associated with BC risk including the work of iCOGS^4,5^ and subsequent replication studies in different populations^6–11^; however, these results were still not enough to explain the hereditary of BC.

Matrix metalloproteinases (MMPs) are a group of zinc-dependent endopeptidases that can breakdown almost all extracellular matrix components^12^. MMPs are known to play a crucial role in tumour invasion and metastasis and are also involved in a series of other cellular process^13^. Previous studies have reported that aberrant MMP expression is associated with risk of several types of cancers^14,15^ and suggested that serum MMP level could be used as biological markers for early detection of disease^16^. Decock J *et al*.^17^ reported high expression levels of *MMP2* and *MMP11* as well as interactions of *MMP2*-*MMP10* and *MMP8*-*MMP9* in patients with BC. The enzyme MMP8 could degrade the type I, II and III collagens, while MMP9 degrades type IV and V collagens. *MMP8* and *MMP9* genes have multiple transcript variants, which have been associated with BC risk in European and American populations, especially advanced stage, lymph node metastasis and poor prognosis^18,19^. However, the specific pathogenesis is still unclear. Additionally, few previous studies focused on the polymorphisms of *MMP8* and *MMP9* in Chinese Han BC patients. We intended to identify more SNPs in *MMP8* and *MMP9* gene that may be associated with BC risk.

The SNPs rs3740938, rs2012390 and rs11225394 were reported to be associated with the risk of osteonecrosis of the femoral head in the Chinese Han population^20,21^. Rs1940475 may take part in modifying the host response to inflammatory stimuli^22^. Rs11225395 and rs2274755 were considered as candidate SNPs in previous association studies on bladder cancer and glaucoma^23,24^. In addition, the haplotype constructed by rs3918249, rs3918254 and rs3787268 in MMP-9 were associated with primary angle-closure glaucoma in a Chinese Han population^25^. In this case-control study, we selected and genotyped the above nine SNPs in *MMP8* and *MMP9* to evaluate their association with risk of BC.

## Results

A total of 571 BC patients and 578 healthy controls were recruited in the study. The ages of the patient and control groups are described in Table 1. The mean age of the participants was 50.91 years in the case group and 50.21 years in the control group. There was no significant difference in the distribution of age between BC patients and healthy controls (*P* > 0.05).

**Table 1:** Basic characteristic of patients with breast cancer and the control individuals.

Characteristics	Cases (N = 571)	Controls (N = 578)	*P*
Age			0.517
Mean ± SD	50.91 ± 11.23	50.21 ± 10.11	

SD: Standard deviation; *P* value was calculated by Welch’s t test.

The basic information of the *MMP8* and *MMP9* polymorphisms (rs3740938, rs2012390, rs1940475, rs11225394, rs11225395, rs3918249, rs2274755, rs3918254 and rs3787268) is shown in Table 2, including gene, band, position, role, alleles and minor allele frequency (MAF). One SNP (rs2274755) was excluded due to significant deviation from Hardy-Weinberg equilibrium (*P* < 0.05). By comparing the difference of allele frequencies between two groups, we found that the frequency of the “A” allele of rs3787268 was significantly lower in BC cases than in controls (32.2% versus 37.7%), which suggested that the “A” allele of rs3787268 may be associated with a decreased risk of BC [odds ratios (ORs) = 0.822, and 95% confidence intervals (CIs): 0.692–0.975, *P* = 0.025].

**Table 2:** Basic information of candidate SNPs in this study.

SNP ID	Gene	Band	Position	Role	Alleles A/B	*p*-HWE	MAF		*P* ^a^	OR (95% CI)
							Case	Control		
rs3740938	*MMP8*	11q22.2	102587062	Coding exon	A/G	0.794	0.232	0.199	0.057	1.214 (0.994–1.481)
rs2012390	*MMP8*	11q22.2	102590777	Intron	G/A	0.349	0.264	0.231	0.063	1.197 (0.990–1.447)
rs1940475	*MMP8*	11q22.2	102593248	Coding exon	T/C	0.713	0.380	0.343	0.068	1.172 (0.988–1.390)
rs11225394	*MMP8*	11q22.2	102595413	Intron (boundary)	T/C	0.065	0.118	0.115	0.813	1.031 (0.799–1.332)
rs11225395	*MMP8*	11q22.2	102596480	Promoter	A/G	0.514	0.366	0.335	0.116	1.147 (0.966–1.362)
rs3918249	*MMP9*	20q13.12	44638136	Intron	T/C	1	0.325	0.307	0.345	1.089 (0.913–1.298)
rs2274755	*MMP9*	20q13.12	44639692	Intron (boundary)	T/G	0.001^#^	0.147	0.121	0.067	1.252 (0.984–1.593)
rs3918254	*MMP9*	20q13.12	44640391	Intron (boundary)	T/C	0.579	0.184	0.183	0.960	1.006 (0.814–1.242)
rs3787268	*MMP9*	20q13.12	44641731	Intron variant	A/G	0.184	0.332	0.377	0.025*	0.822 (0.692–0.975)

SNP: single-nucleotide polymorphism, Alleles A/B: Minor/major alleles; MAF, minor allele frequency; OR: odds ratio, CI: confidence interval, HWE: Hardy–Weinberg equilibrium;

^#^HWE *p*-value < 0.05 was excluded;

^a^*p* values were calculated using two-sided Chi-squared test;

*p < 0.05 indicates statistical significance.

Next, we investigated the association between each SNP and risk of BC based on different genetic models (Table 3). When the sum of AIC and BIC were at the minimum, the model was considered as the best model. Under the best model-recessive model, the minor allele “T” of rs11225394 in *MMP8* was associated with an increased risk of BC (OR = 3.94, 95% CI: 1.10–14.07, *P* = 0.019). Under the best model-dominant model, the minor allele “A” of rs3787268 was associated with decreased risk of BC (OR = 0.74, 95% CI: 0.59–0.94, *P* = 0.014).

**Table 3:** Genotype frequencies of the SNPs and their associations with risk of breast cancer.

SNP	Model	Genotype	Case	Control	Without adjustment				With adjustment of age			
					OR (95% CI)	*P*	AIC	BIC	OR (95% CI)	*P*	AIC	BIC
rs11225394	Codominant	C/C	445 (78.5%)	445 (77.5%)	1	0.033*	1580.9	1596	1	0.042*	1576.2	1596.4
		C/T	110 (19.4%)	126 (21.9%)	0.87 (0.65–1.16)				0.88 (0.66–1.17)			
		T/T	12 (2.1%)	3 (0.5%)	4.00 (1.12–14.26)				3.83 (1.07–13.71)			
	Dominant	C/C	445 (78.5%)	445 (77.5%)	1	0.7	1585.6	1595.6	1	0.71	1580.4	1595.5
		C/T-T/T	122 (21.5%)	129 (22.5%)	0.95 (0.71–1.25)				0.95 (0.72–1.26)			
	Recessive	C/C-C/T	555 (97.9%)	571 (99.5%)	1	0.015*	1579.8	1589.8	1	0.019*	1575	1590.1
		T/T	12 (2.1%)	3 (0.5%)	4.12 (1.16–14.65)				3.94 (1.10–14.07)			
	Log-additive	—	—	—	1.03 (0.80–1.33)	0.81	1585.7	1595.7	1.03 (0.80–1.33)	0.81	1580.5	1595.6
rs3787268	Codominant	G/G	253 (44.3%)	216 (37.5%)	1	0.058	1590.4	1605.5	1	0.043*	1584.5	1604.7
		A/G	257 (45%)	286 (49.6%)	0.77 (0.60–0.98)				0.76 (0.59–0.97)			
		A/A	61 (10.7%)	74 (12.8%)	0.70 (0.48–1.03)				0.69 (0.47–1.01)			
	Dominant	G/G	253 (44.3%)	216 (37.5%)	1	0.019*	1588.6	1598.6	1	0.014*	1582.8	1597.9
		A/G-A/A	318 (55.7%)	360 (62.5%)	0.75 (0.60–0.95)				0.74 (0.59–0.94)			
	Recessive	G/G-A/G	510 (89.3%)	502 (87.2%)	1	0.26	1592.8	1602.9	1	0.22	1587.3	1602.4
		A/A	61 (10.7%)	74 (12.8%)	0.81 (0.57–1.16)				0.80 (0.55–1.14)			
	Log-additive	—	—	—	0.82 (0.68–0.97)	0.022*	1588.8	1598.9	0.81 (0.68–0.96)	0.016*	1583	1598.1

ORs: odds ratios; CI: confidence interval; AIC: Akaike’s Information criterion; BIC: Bayesian Information criterion.

*p value < 0.05 indicates statistical significance.

**Figure 1:**
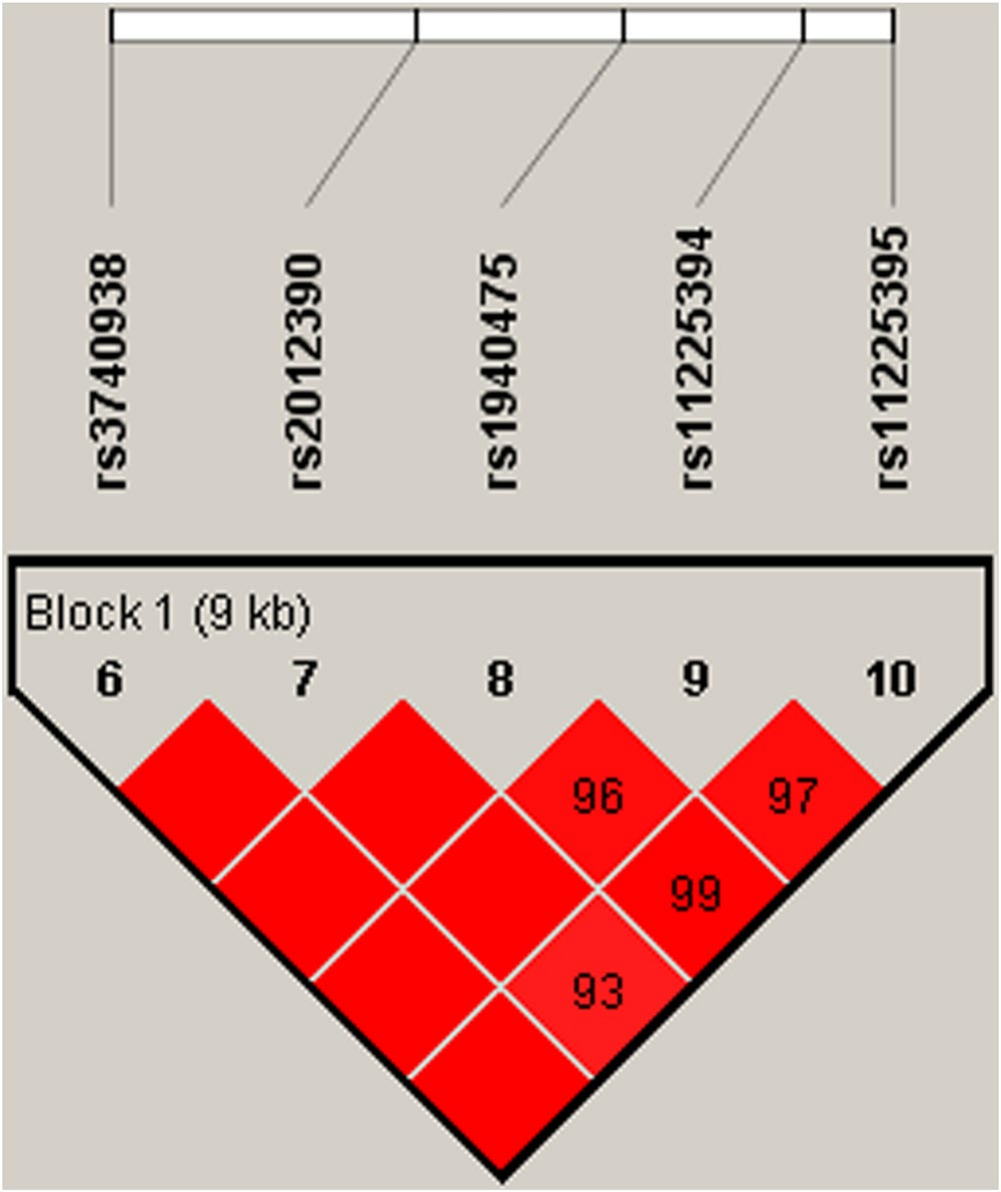
D′ linkage map for the five SNPs in *MMP8*.

**Figure 2:**
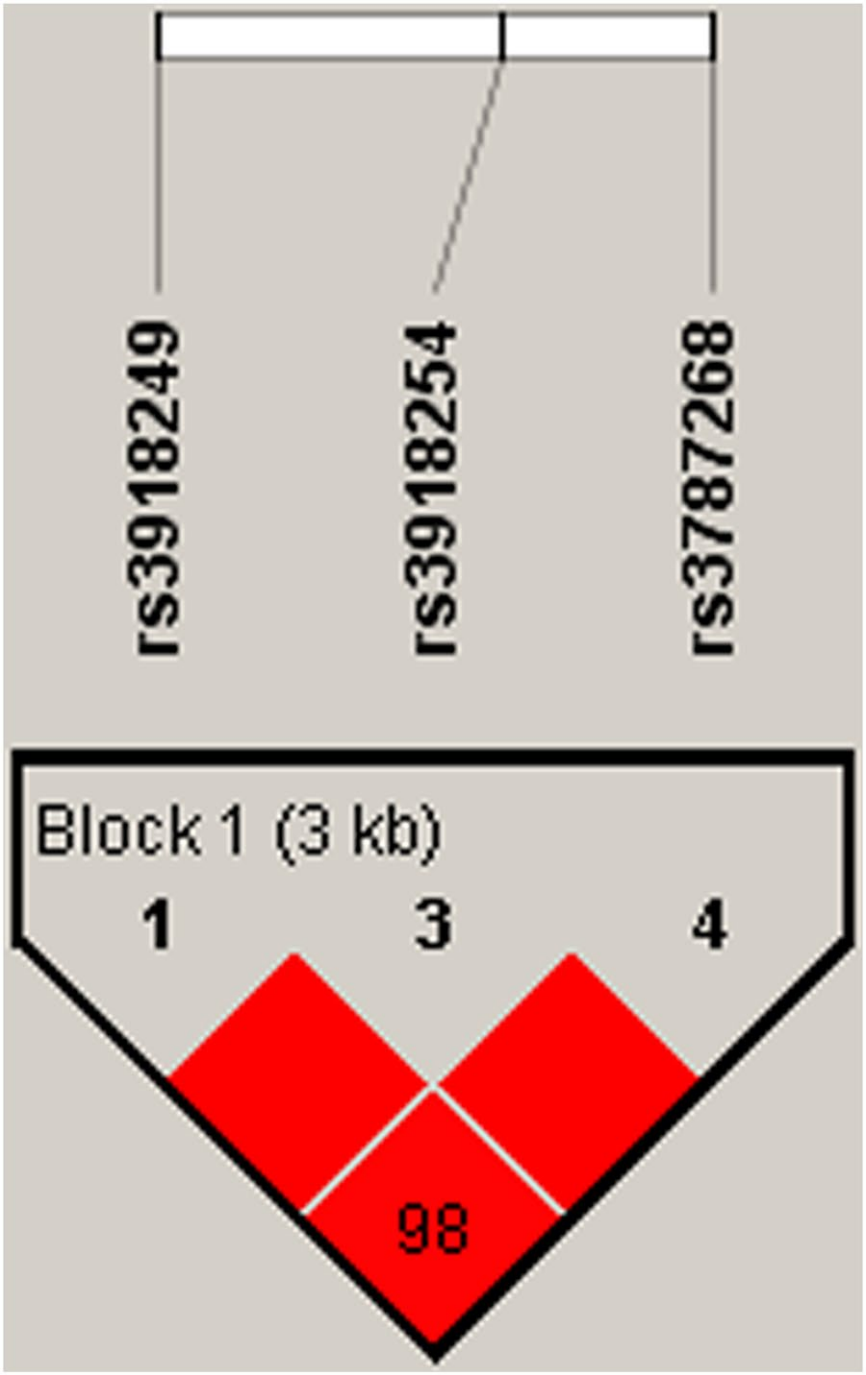
D’ linkage map for the three SNPs in *MMP9*.

The association of *MMP8* and *MMP9* haplotypes with the risk of BC was analysed. Figures 1 and 2 show the linkage disequilibrium (LD) block in *MMP8* and *MMP9*. The association between different haplotypes and BC risk is shown in Table 4. The haplotype “AGTCA” constructed by rs3740938, rs2012390, rs1940475, rs11225394, and rs11225395 was associated with an increased risk of BC after the adjustment (OR = 1.23; 95% CI = 1.00–1.51; *P* = 0.048). The haplotype “CCG” constructed by rs3918249, rs3918254 and rs3787268 was also associated with an increased risk of BC after the adjustment (OR = 1.37; 95% CI = 1.05–1.77; *P* = 0.019).

**Table 4:** *MMP8* and *MMP9* haplotype frequencies and the association with the breast cancer risk.

Blocks	Genes	SNPs	Haplotype	Freq-case	Freq-control	Without adjustment		With adjustment of age	
						OR (95% CI)	*P*	OR (95% CI)	*P*
1	*MMP8*	rs3740938|rs2012390|rs1940475|rs11225394|rs11225395	GACCG	0.617	0.654	1.00	—	1.00	—
			AGTCA	0.232	0.200	1.22 (1.00–1.50)	0.051	**1**.**23 (1**.**00–1**.**51)**	0.048*
			GATTA	0.116	0.112	1.09 (0.83–1.42)	0.530	1.09 (0.84–1.43)	0.520
			GGTCA	0.018	0.022	0.88 (0.50–1.55)	0.660	0.90 (0.51–1.59)	0.710
			GGTCG	0.014	0.010	1.51 (0.71–3.22)	0.280	1.51 (0.71–3.23)	0.280
2	*MMP9*	rs3918249|rs3918254|rs3787268	CCA	0.331	0.375	1.00	—	1.00	—
			TCG	0.325	0.305	1.21 (0.99–1.48)	0.064	1.22 (0.99–1.49)	0.060
			CTG	0.185	0.183	1.16 (0.91–1.48)	0.240	1.19 (0.93–1.52)	0.170
			CCG	0.158	0.134	**1**.**34 (1**.**04–1**.**74)**	0.025*	**1**.**37 (1**.**05–1**.**77)**	0.019*

Freq: frequency; ORs: odds ratios; CI: confidence interval; * *p* value < 0.05 indicates statistical significance.

Finally, we summarized the OR and p-values of candidate SNP from previous studies. The results are listed in Table 5.

**Table 5:** The OR and 95% CI of candidate SNPs in previous studies.

SNPs	Minor/Major allele	OR(95% CI)	*P*	Disease	Reference
rs3740938	A/G	1.36 (1.02–1.82)	0.034	Steroid-induced osteonecrosis of the femoral head	20
rs2012390	G/A	1.55 (1.10–2.21)	0.013		
rs11225395	A/G	1.34 (1.04–1.73)	0.023		
rs11225394	T/C	1.44 (1.05–1.96)	0.023	Osteonecrosis of the femoral head	21
rs11225395	T/C	0.96 (0.63–1.46)	0.836	Bladder cancer	23
rs2274755	T/G	1.67 (1.12–2.50)	0.021	Glaucoma	24
rs3918249	T/C	1.041 (0.477–2.269)	0.010	Primary angle-closure glaucoma	25
rs3918254	T/C	4.397 (1.455–13.289)	0.006		
rs3787268	A/G	1.009 (0.764–1.334)	0.004		

## Discussion

In this study, we investigated the associations between nine candidate SNPs in *MMP8* and *MMP9* genes and BC risk in a Chinese Han population. We found that rs11225394 in *MMP8* and rs3787268 in *MMP9* are significantly associated with BC risk. We also found two haplotypes were associated with increased risk of BC: haplotype “AGTCA” constructed by rs3740938, rs2012390, rs1940475, rs11225394, and rs11225395 in *MMP8*, and haplotype “CCG” constructed by rs3918249, rs3918254 and rs3787268 in *MMP9*. Our results suggest that the polymorphisms of *MMP8* and *MMP9* may play an important role in the risk of BC in the Chinese Han population.

MMPs are a family of endopeptidases involved in the degradation of extracellular matrix and basement membrane barriers. As such, they contribute when tumour cells separate from adjacent normal tissues^26,27^. Several studies have revealed that MMPs are associated with the invasion and metastasis of tumour cells. MMP8 and MMP9 are members of the MMPs family. Plasma MMP8 levels were lower in BC patients than in healthy control individuals, and MMP8 was found to protect against lymph node metastasis in BC patients^28^. Differential expression of MMP9 was found in BC cells with different degrees of cellular differentiation^29^. We identified two SNPs in *MMP8* and *MMP9* that were associated with risk of BC, which further corroborates the association between MMPs and BC risk.

In the present study, we investigated nine SNPs in the *MMP8* and *MMP9* genes. Among these SNPs, rs3740938, rs2012390, rs11225394, rs2274755 and rs3918254 were identified that have been associated with risk of osteonecrosis of the femoral head in the Chinese Han population^20,21,30^. We report for the first time that rs11225394 was associated with BC risk. This association needs to be confirmed in a further study with a larger sample size. Rs1940475 was found to have association with community-acquired pneumonia-associated sepsis and inflammatory response in Caucasians^22,31^. Rs11225395 was associated with bladder cancer risk in a Polish population^23^. Rs3918249 was demonstrated to be associated with risk for primary angle-closure glaucoma in Chinese Han population^25^ and risk for asthma in Mexican paediatric patients^32^. Rs3787268 was also correlated with BC prognosis in previous studies; the “GA” and “AA” genotypes of rs3787268 were significantly associated with 1.52-fold risk of BC in Native American women^33^. However, in our study, we found that the minor allele “A” of rs3787268 was associated with decreased risk of BC in Chinese women, which is inconsistent with previous result. This inconsistency may be due to the limited sample size and different populations.

Our study has some intrinsic limitations. First, the sample size was relatively small, so the results identified here just provide some pilot data for continued in-depth studies on BC. Second, the occurrence of BC was also influenced by BMI, menarche, marital status, parity, and so on. We failed to collect these specific data, which is a weakness of the study. Additionally, although we identified significant SNPs and haplotypes with risk of BC, this is still not enough to explain the molecular mechanism between MMP and the onset and development of BC.

In conclusion, the present study showed that polymorphisms of *MMP8* and *MMP9* are associated with risk of BC in a Chinese Han population. Further studies will focus on two directions. One is to demonstrate the association between MMP and BC risk in larger sample sizes and different populations; the other is to investigate the mechanisms or pathways by which MMP influence BC risk.

## Materials and Methods

### Study participants

A total of 571 BC patients and 578 healthy controls were consecutively recruited between June 2012 and July 2016 at the First Affiliated Hospital of Xi’an Jiaotong University, People’s Republic of China. Patients diagnosed with other types of cancers or who underwent radiotherapy or chemotherapy were excluded. The controls with no history of cancer were recruited from the physical examination centre of our hospital.

### SNP selection and genotyping

Nine SNPs (rs3740938, rs2012390, rs1940475, rs11225394, rs11225395, rs3918249, rs2274755, rs3918254 and rs3787268) in *MMP8* and *MMP9* were selected from previous association studies. DNA extraction and concentrations were done as previously described^34^. SNP genotyping was performed by the Sequenom MassARRAY RS1000, and the data analyses were completed by Sequenom Type 4.0. The polymerase chain reaction (PCR) primers used for the study are listed in Table 6.

**Table 6:** Primers used in this study.

SNPID	First PCR primer	Second PCR primer	UEP SEQ
rs3740938	ACGTTGGATGGTCAGTAAGAGGAATCAAAG	ACGTTGGATGTGACATTTGATGCTATCAC	GATGCTATCACCACACT
rs2012390	ACGTTGGATGACTGTTTCTAGGTCACACCC	ACGTTGGATGTCAGGGAGAGGAAGCAATTC	gAAGCAAATGTGAGGAAGAT
rs1940475	ACGTTGGATGTTTGGGTTGAATGTGACGGG	ACGTTGGATGTAAAACCACCACTGTCAGGC	CTCCACAGCGAGGCTTTT
rs11225394	ACGTTGGATGCAATCTCAAACTAATCACCC	ACGTTGGATGTTAGGAAATAGTGTGGGTTG	AGTGTGGGTTGTTTTCTCTT
rs11225395	ACGTTGGATGAGAGCTGCTGCTCCACTATG	ACGTTGGATGGTTTAGAGAGACTGAGCTGG	gCTGAGCTGGGAGCTACTATA
rs3918249	ACGTTGGATGAAGCACTGGTGTCTGGAAAG	ACGTTGGATGGATTACAAGTGTGAGCCGTC	gaaGTCATGCCCAGCAGGGACTA
rs2274755	ACGTTGGATGGGGAGAGAATGAAGGGAATC	ACGTTGGATGTTCGACGATGACGAGTTGTG	gCTGGGCAAGGGCGTCGGT
rs3918254	ACGTTGGATGTCTTCGGCTTCTGCCCGAC	ACGTTGGATGCAATACATGATGAGAGGGCG	CTGGTAGACAGGGTGGA
rs3787268	ACGTTGGATGATCCTGGGCCATAGAGGATG	ACGTTGGATGCTTCCCAAACCACAGGACTT	aCACAGGACTTTCTTCTTCTTCTTTTT

UEP SEQ, unextended mini‐sequencing primer.

### Statistical Analyses

All of the statistical analyses were performed with Microsoft Excel (Microsoft Corporation, Redmond, WA, USA) and the SPSS 21.0 statistical package (SPSS, Chicago, IL, USA). SNP allele frequencies in the control subjects were tested for departure from Hardy–Weinberg Equilibrium (HWE) before analysis. Differences in the distributions of allele frequencies between the cases and controls were evaluated using chi-square tests. Four models (co-dominant, dominant, recessive, and log-additive) were used to assess the association between each genotype and the BC risk^35^. Akaike’s Information criterion (AIC) and Bayesian Information criterion (BIC) were used to select the best model for each SNP. ORs and 95%CIs were calculated with and without adjustment for age. All *p* values presented in this study were two sided, and *p* = 0.05 was considered the cutoff for statistical significance.

Haploview software version 4.2 was used to analyse the association between haplotypes and BC. Linkage disequilibrium (LD) analysis was performed using genotype data from all the subjects. Statistical significance was established when *p* < 0.05.

### Approval

All of the participants provided written informed consent. The Human Research Committee for Approval of Research Involving Human Subjects, the First Affiliated Hospital of Xi’an Jiaotong University, approved the use of human blood samples in this study. All methods were performed in accordance with the relevant guidelines and regulations.

## Data Availability

The datasets generated during and/or analysed during the current study are available from the corresponding author on reasonable request.
